# Comparison of Semi-Quantitative Scoring and Artificial Intelligence Aided Digital Image Analysis of Chromogenic Immunohistochemistry

**DOI:** 10.3390/biom12010019

**Published:** 2021-12-23

**Authors:** János Bencze, Máté Szarka, Balázs Kóti, Woosung Seo, Tibor G. Hortobágyi, Viktor Bencs, László V. Módis, Tibor Hortobágyi

**Affiliations:** 1 Division of Radiology and Imaging Science, Department of Medical Imaging, Faculty of Medicine, University of Debrecen, 4032 Debrecen, Hungary; bencze.janos@med.unideb.hu; 2 ELKH-DE Cerebrovascular and Neurodegenerative Research Group, Department of Neurology, University of Debrecen, 4032 Debrecen, Hungary; 3 Horvath Csaba Laboratory of Bioseparation Sciences, Research Center for Molecular Medicine, Faculty of Medicine, University of Debrecen, 4032 Debrecen, Hungary; szarka.mate@med.unideb.hu; 4 Vitrolink Kft., 4033 Debrecen, Hungary; ktibalazs@vitrolink.com; 5 Institute for Nuclear Research, 4026 Debrecen, Hungary; 6 Department of Surgical Sciences, Radiology, Uppsala University, 751 85 Uppsala, Sweden; wooah.seo@gmail.com; 7 Institute of Pathology, Albert Szent-Györgyi Medical School, University of Szeged, 6725 Szeged, Hungary; tibor4hortobagyi@googlemail.com; 8 Department of Neurology, Faculty of Medicine, University of Debrecen, 4032 Debrecen, Hungary; bencsv95@gmail.com; 9 Department of Behavioural Sciences, Faculty of Medicine, University of Debrecen, 4032 Debrecen, Hungary; laszlo.modis@gmail.com; 10 Department of Old Age Psychiatry, Institute of Psychiatry Psychology and Neuroscience, King’s College London, London SE5 8AF, UK; 11 Centre for Age-Related Medicine, SESAM, Stavanger University Hospital, 4011 Stavanger, Norway

**Keywords:** artificial intelligence (AI), digital image analysis, immunohistochemistry, semi-quantitative scoring

## Abstract

Semi-quantitative scoring is a method that is widely used to estimate the quantity of proteins on chromogen-labelled immunohistochemical (IHC) tissue sections. However, it suffers from several disadvantages, including its lack of objectivity and the fact that it is a time-consuming process. Our aim was to test a recently established artificial intelligence (AI)-aided digital image analysis platform, Pathronus, and to compare it to conventional scoring by five observers on chromogenic IHC-stained slides belonging to three experimental groups. Because Pathronus operates on grayscale 0-255 values, we transformed the data to a seven-point scale for use by pathologists and scientists. The accuracy of these methods was evaluated by comparing statistical significance among groups with quantitative fluorescent IHC reference data on subsequent tissue sections. The pairwise inter-rater reliability of the scoring and converted Pathronus data varied from poor to moderate with Cohen’s kappa, and overall agreement was poor within every experimental group using Fleiss’ kappa. Only the original and converted that were obtained from Pathronus original were able to reproduce the statistical significance among the groups that were determined by the reference method. In this study, we present an AI-aided software that can identify cells of interest, differentiate among organelles, protein specific chromogenic labelling, and nuclear counterstaining after an initial training period, providing a feasible and more accurate alternative to semi-quantitative scoring.

## 1. Introduction

Digital technology is an organic part of our daily lives and has a huge impact on our profession, social network, and leisure activities. It has had an impact in nearly every aspect of modern medicine, but the degree of digitalization is highly uneven among medical fields [1]. While conventional light microscopy is still the gold standard method for investigations in the pathological workup, in radiology, a new discipline called radiomics has recently evolved, integrating the work of radiologists, software engineers, and data scientists [2]. However, in pathology, the application of digital image analysis, especially aided by artificial intelligence (AI), is still relatively rare [3]. Immunohistochemistry (IHC) is a fundamental technique that is used to identify certain antigens in tissue sections with diagnostic, differential diagnostic and prognostic value [4,5,6]. The chromogen 3,3′-Diaminobenzidine (DAB) is widely-used to visualize proteins of interest. However, there is no stochiometric relationship between the chromogen’s intensity and the quantity of antigens; in a standardized experiment, a stronger DAB intensity indicates a higher protein level in the tissue and vice versa [7]. IHC intensity scoring is a method that is widely used for the assessment of protein quantity and is usually ranked on a four-point scale (0, 1, 2, 3) in pathological diagnostics and research [8,9,10,11,12]. However, this semi-quantitative technique is subjective and highly inaccurate and demonstrates significant intra- and inter-observer variability [13]. One possible solution is the application of machine learning, which is valuable in automating workflows where repetitive, lengthy, and monotonous tasks are encountered [14]. In histopathology the task at hand is the qualitative and quantitative assessment of differentially stained cellular morphology of tissue sections. On the differentially stained samples, the major organelles of cells can be distinguished with different colours due to their unique chemical interactions with the dye molecules. Parameters describing the shape of cells, optical densities, etc., differ from type to type. Consequently, cells (i.e., neurons) can be identified by the ‘old-fashioned’ manual way looking of into the microscope or via the highly automated processing of digital images (taken by microscope cameras or slide scanners) using different analytical algorithms. Moreover, AI-aided platforms cannot only recognize different tissue structures, but they can also measure staining intensity more accurately than semi-quantitative scoring systems can [15]. In this study, we examine the usability of AI-aided digital image analysis to estimate the protein levels on DAB-labelled IHC slides. Furthermore, we also investigated the reliability of the software by comparing its intensity results to the semi-quantitative IHC scores of three experimental groups that have been assessed by five scientists. The inter-observer variability of the conventional scoring method was also evaluated. The current research focused strictly on the comparison of the two methods. The biological significance of the labelled protein’s (lemur tyrosine kinase 2 (LMTK2)) was previously published in an immunofluorescent IHC study [16]. Furthermore, we declare that this is the first time where chromogenic (CHR)-IHC intensity scoring results and this type of investigation have been published.

## 2. Materials and Methods

### 2.1. Sample Selection and Processing

Post-mortem formalin-fixed paraffin-embedded (FFPE) human brain samples were obtained from the Medical Research Council (MRC) London Neurodegenerative Diseases Brain Bank at the Institute of Psychiatry, Psychology and Neuroscience, King’s College London. All procedures were conducted under the ethical approval of the Institutional Ethics Committee of the MRC London Neurodegenerative Diseases Brain Bank (18/WA/0206) at the Institute of Psychiatry, Psychology and Neuroscience, King’s College London, and the Brains for Dementia Research Project (08/H0704/128+5). Informed consent for autopsy, neuropathological assessment, and research participation were obtained for all subjects, and data were anonymized. Block taking, immunohistochemical labelling, and neuropathological assessment for neurodegenerative diseases were carried out in accordance with standard protocols as described in detail in earlier studies [6,16,17].

We established three groups, including two different neurodegenerative dementias (Alzheimer’s disease and Dementia with Lewy bodies) with severe neuropathological stages and age-matched controls (CNT) with no known neurodegenerative condition. The assessed brain region was the middle frontal gyrus (Brodmann area 9). There were 6-6 (n = 18 in total) samples in the three experimental groups. CHR-IHC labelling of LMTK2 was performed according to a standardized protocol that had been published earlier [16].

The slides were scanned with a Virtual Slide Microscope VS120 (Olympus Corp., Tokyo, Japan) with the same illumination intensities, exposure times, and camera settings. Focusing on the cortex, 39 photos/case were taken from the WSI files at medium magnification (200×), covering the whole cortical area on each slide. Both digital image analysis and semi-quantitative scoring were performed on these photos to guarantee that the software and the observers evaluated the same images and to avoid discrepancies resulting from the different settings of the light microscope and the slide scanner as well as the digital display.

### 2.2. IHC Intensity Scoring

**Figure 1:**
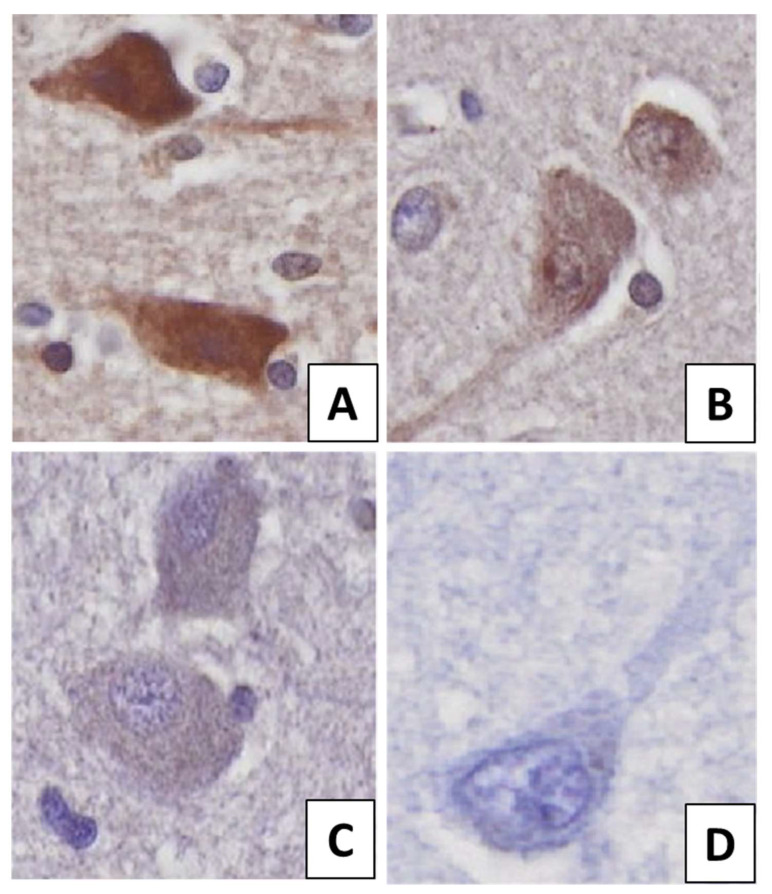
The different immunolabelling intensities in neurons: (**A**) strong positivity (3+), (**B**) moderate positivity (2+), (**C**) mild positivity (1+), (**D**) negative (0). The protein was visualized by 3,3′-Diaminobenzidine (DAB) chromogen. Nuclear counterstain with haematoxylin.

Semi-quantitative cellular scoring was carried out using a four-point scale based on the IHC intensity of the cells: negative (0), mild positivity (1+), moderate positivity (2+), and strong positivity (3+) (Figure 1). A few reference images were analysed quantitatively with ImageJ software (National Institute of Health, Bethesda, MD, US), using Cell counter module to calculate the exact average IHC intensity score of the given images. These were used as reference images and allowed the investigators to execute a more accurate semi-quantitative scoring methodology such as the one described in our previous work [4]. Then, the observers determined the mean IHC intensity scores for each image. To do this, we extended the original scores with values of 0.5, 1.5, and 2.5, e.g., if an image contained mild (1+) and moderate (2+) positive cells in a ratio that was approximately 1:1, then we assigned a score of 1.5. Thus, the final scores of the images were determined on a 7-point scale.

### 2.3. Digital Image Analysis

The core of the image analysis software of Pathronus platform (V1.2, Vitrolink Kft., Debrecen, Hungary) was a Convolutional Neural Network (CNN) [18]. Pathronus was developed as an AI-assisted image analysis platform that was only to be used for the purposes of research and development. It combines the concept of a pathology-focused online shared workspace with state-of-the-art CNN techniques. In general, the two aspects of the application work to improve each other in a feedback-loop system: The users provide new images that are uploaded and analysed to determine whether the analysed objects were detected correctly by the AI or whether points of interest should be annotated manually, and these images act as new training data for the network. This improves the capabilities and accuracy of the CNN, allowing it to make better predictions when a new image is uploaded later on. Since the platform functions use diverse user images (and equipment), most of the biases that a network might naturally develop when trained using data from a handful of sources can be naturally eliminated. With a sufficient quantity of training data, such a system might be capable of identifying any desirable features on any type of pathological imagery.

**Figure 2:**
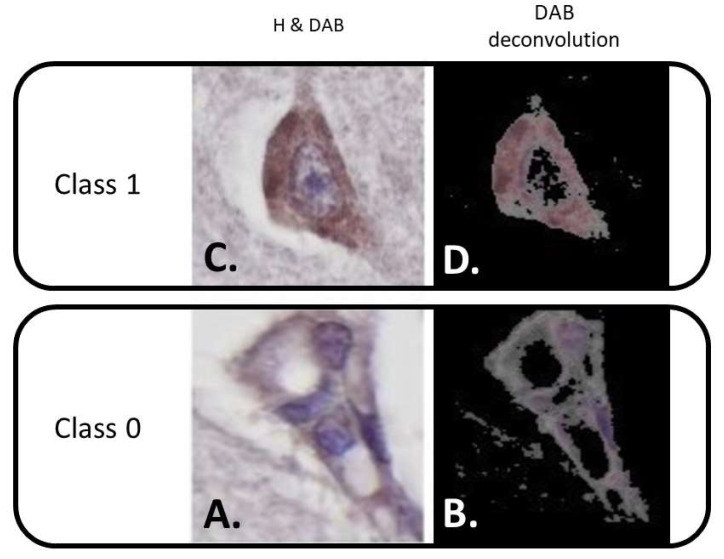
The selection criteria (**A**,**C**) and the deconvoluted 3,3′-Diaminobenzidine (DAB) chromogen (**B**,**D**). Class 0 represents an example of a misidentified item (vessels which mimic the shape of a neuron). Class 1 depicts an ideal neuron that could be used for the intensity measurements, which has a large amount of cytoplasm and an easily observed nucleus. H = nuclear counterstain; haematoxylin.

In this particular case, the constructed convolutional network was trained on thousands of neurons that had been gathered into two morphology classes to detect specific IHC patterns. As the starting step, the recognition criteria were first determined, which were the abundant cytoplasm and the large, well-recognized nucleus that was visible in the given section plane as the types of accepted neurons (Class 1, Figure 2C). Any other object not falling into the previously mentioned categories was labeled as a rejected item (Class 0, Figure 2A) for the purposes of the training process. A confusion matrix analysis was performed to test the accuracy of the system.

By design, the Pathronus platform runs an object detection algorithm, which feeds images to the CNN and marks the areas of the morphology class as a Region of Interest (ROI) in the images (e.g., specified the exact number, coordinates, and extent of the neurons in the images of the tissue sections). The AI model system that was used in this research is based on the Keras-RetinaNet module. It implements deep learning and uses an algorithm called RetinaNet, which is one of the most advanced object recognition algorithms that is used to detect features in images. The code itself is based on the Keras deep learning framework that is part of the python programming language [19]. As a result, it successfully recognized and cropped Class 1 DAB-stained neurons in the images (Figure 2D). The neurons were manually checked by humans. The accepted neurons were found on the same set of images that the pathologist used for scoring and were processed for DAB intensity signal levels by the platform. It used colour deconvolution with the same parameters applied to all ROIs to separate the nuclear counterstain haematoxylin and the cytoplasmic DAB signals in all of the cropped neurons (see Figure 2). The platform only kept the inverted DAB signal and measured the average intensity (8-bit grayscale) of all of the neurons with the same settings (Figure 2D). In an 8-bit image, 0 represents black and 255 means white, thus for a better graphical presentation and to avoid misunderstandings, we used an inverse grayscale, where the more intense (darker) DAB signals have higher inverse grayscale values (i.e., darkest value = 255). The individual intensity values of the images corresponding to the CNT, AD, and DLB cases were then summarized. Finally, we determined the mean inverse gray intensities of the experimental groups. Please note that the analysis focused on the neuron intensity that had been identified by the software and that had been further evaluated by the pathologists participating this study; therefore, the surpassing performance of the neural network module was not a fundamental requirement, as the neurons that were subjected to intensity analysis were manually checked and filtered prior to assessment. It was not within the scope of this study to create an all-out version of a neuron classifier software. 

### 2.4. Comparison between Semi-Quantitative Scoring and Digital Image Analysis

Because original semi-quantitative scoring is based on a four-point scale while the software used the grayscale ranges from 0 to 255, it was not possible to compare the methods directly. Therefore, we converted the inverse grayscale values of the neurons into IHC intensity scores according to the following formula: 0–33➔0; 34–107➔1; 108–181➔2; 182–255➔3, where the first ranges refer to the inverse grayscale values and where the numbers after arrows are the converted scores. The first inverse grayscale range (0–33) was derived from the digital image analysis of the IHC negative, haematoxylin-only slide, where the maximum measured value was 33, and the rest of the conversion resulted from the division of the grayscale range of 34–255 into three equal parts. Then, we calculated the mean scores of the images that were from cell-level data. In order to perform a valid inter-rater reliability analysis, a second conversion to the 7-point scale was applied on the mean values based on the following formula: 0–0.25➔0; 0.26–0.75➔0.5; 0.76–1.25➔1; 1.26–1.75➔1.5; 1.76–2.25➔2; 2.26–2.75➔2.5; 2.75–3➔3. Through this formula, the IHC intensity data that were measured by the Pathronus platform and the semi-quantitative scores that were determined by the observers are easily comparable. Nevertheless, it should be noted that the presentation of digital image analysis results on a seven-point scale due to double-conversion decreases the evaluation accuracy by losing a significant amount of resolution.

### 2.5. Statistical Analysis

Inter-observer reliability for the experimental groups was investigated by the comparison of the intensity scores of the images (n = 234/group) given by the observers and Pathronus using Fleiss’ kappa. Pairwise comparisons of the observers (including Pathronus) were also performed with Cohen’s kappa. Strength of agreement was adopted from the study by Landis and Koch [20] as follows: *κ <* 0.20➔Poor; 0.21–0.40➔Fair; 0.41–0.60➔Moderate; 0.61–0.80➔Good; 0.81–1.00➔Very good. Statistical tests were executed with IBM SPSS Statistics for Windows, Version 25.0 (IBM Corp., Armonk, NY, USA). To determine the accuracy of the semi-quantitative scoring and original Pathronus, data validation was required. The statistical relevance of the found differences in CHR-IHC intensities among the three experimental groups were calculated for each observer. The Shapiro–Wilk normality test, equal variance test, one-way analysis of variance (ANOVA), and all pairwise comparison (Holm–Sidak method) were carried out using the SigmaPlot 12.0 software (Systat Software Inc., San Jose, CA, USA). We used the previously published quantitative fluorescent IHC analysis on the same disease groups as a reference [16]. 

## 3. Results

The two classes (Figure 2) were populated randomly and represented all cases (CNT, DLB and AD) after annotation was completed utilizing the Pathronus platform. Class 0 consisted of 4061 samples, while Class 1 had 5009 neurons. A total of 70% of the 9070 neurons were used as the training data set, 20 % were for the validation data set, and 10% were used as the test dataset. The confusion matrix that was generated on the test dataset can be seen in Table 1. The total number of test data (for Class 0 and Class 1 summed) was 907. Out of the 907 samples, the AI classified 409 (False Negative (FN) + True Negative (TN)) cells that did not meet the criteria, and 498 (False Positive (FP) + True Positive (TP)) were classified as ideal neurons that were eligible for further intensity processing. In the test dataset, a total of 445 (TN + FP) neurons were determined to be inadequate for further analysis, and 462 (FN + TP) were determined to be ideal neurons. The best performing model reached a total accuracy value of 0.91 and achieved a recall of 0.95, a false positive rate of 0.12, a specificity of 0.87, a precision of 0.88, and a null error rate of 0.49. The misclassification rate (error rate) was 0.08. The loss function return values went from 0.701 (validation) and 0.767 (training) to 0.367 (validation) and 0.278 (training), and a 0.908 validation and a 0.887 training accuracy was obtained by the end of epoch 3996.

The number of neurons on the CHR images that was analysed by Pathronus and that was manually checked by pathologists was 12,516. Supplementary Table S1 contains the summarized inverse mean gray intensities of the images after color deconvolution (see Methods) of the individually processed IHC-stained neurons. The mean intensity values of the individual cases in the experimental groups ranged between 113.58–123.22, 100.21–114, and 107.76–122.8 in CNT, AD, and DLB, respectively. Semi-quantitative scoring was performed by five observers on 39 images/case (n = 234/group). The scores that were given for each group ranged between one and three (Supplementary Table S1). CNT achieved the highest rating, and AD received the lowest scores from the majority of the observers. Double-converted Pathronus values spread from 1 to 2. Table 2 contains the inter-rater reliability among the observers and Pathronus. Sub-tables A, B, and C show the Cohen’s kappa values and the strength of the agreements that were determined in the pairwise comparisons in the CNT, DLB, and AD groups, respectively. Sub-table D includes the overall comparison among the five human observers plus Pathronus using Fleiss’ kappa. Although, Cohen’s kappa values were highly variable among the observers in different groups, poor agreement dominated, while moderate agreement was the least common. Overall agreement with statistically significant (*p* < 0.005) Fleiss’ kappa values was poor in every experimental group.

**Figure 3:**
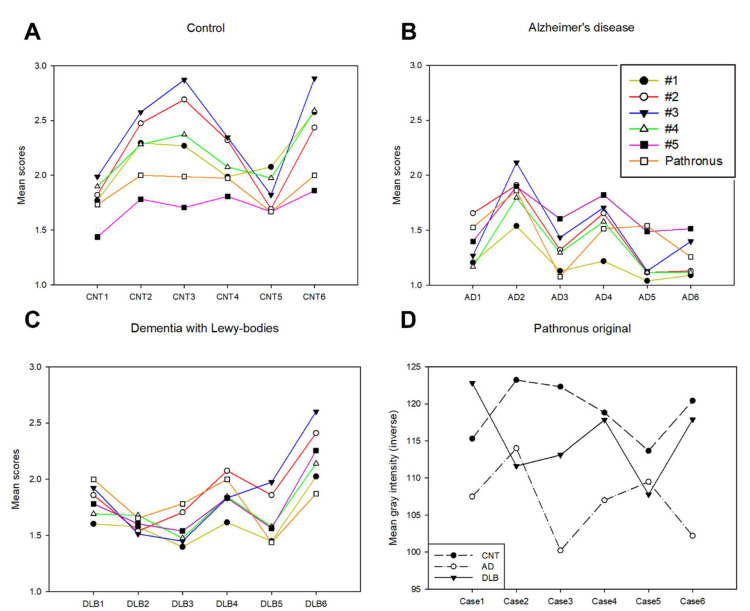
Mean semi-quantitative scores defined by five observers and Pathronus-converted values are depicted on Panels **A**, **B**, **C** for the CNT, AD, and DLB groups, respectively. Panel **D** shows the original Pathronus inverse mean gray intensities of the experimental groups. Insert of panel B specifies the colored lines depicted on panels A, B, and C.; CNT = control; DLB = dementia with Lewy bodies; AD = Alzheimer’s disease; #1–5 = observers].

The statistically significant differences of the distinct experimental groups varied among the observers and between methods. Certain observers (#1 and #4) achieved statistically significant alterations among every group, while others (#2, #3, and #5) did not. However, only the Pathronus analysis (original and converted) was able to reproduce the reference data; specifically, CNT had the strongest and AD had the weakest immunopositivity, and statistically significant differences were revealed between the CNT versus (vs.). AD groups and the DLB vs. AD groups. Figure 3 depicts the summarized intensities of the CNT, AD, and DLB groups by the five observers as well as by the converted (Panels A, B, C) and original Pathronus (Panel D) methods.

## 4. Discussion

**Figure 4:**
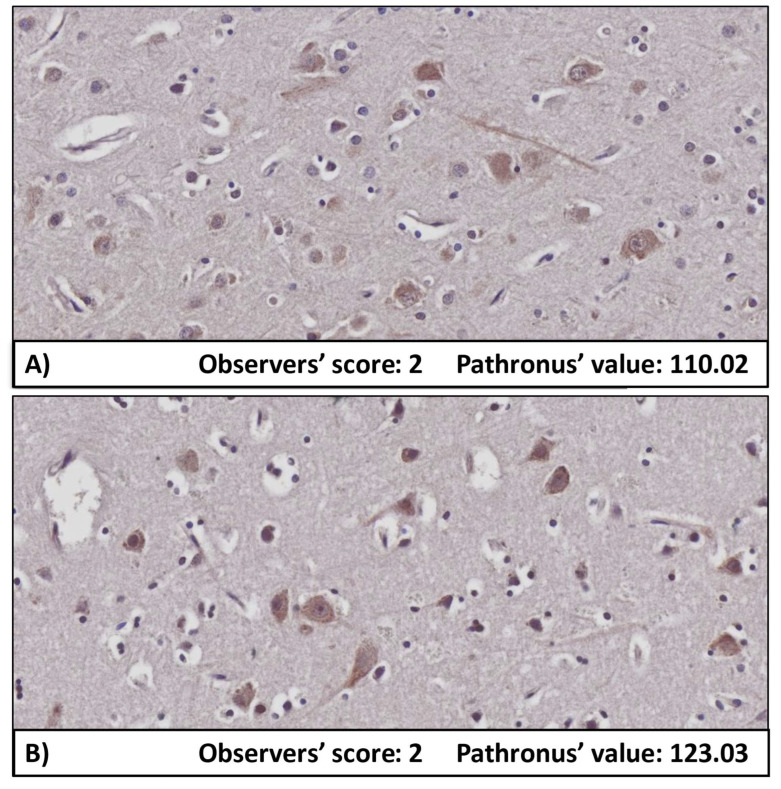
A remarkable limitation of semi-quantitative scoring compared to digital image analysis is the significantly smaller evaluation range (7 vs. 256) due to the difficulties that are experienced when attempting to detect subtle differences in the labelling intensities using the human eye alone. The observers allocated a score of 2 to both of the images, whereas the Pathronus original method revealed that the intensity of Panel **A** was 110.02, while that of Panel **B** was 123.03 on the grayscale (ranged between 0–255). Although the human eye is capable of perceiving small differences, the objective and reproducible categorization of hundreds of images on an extended scale is not possible for human observers whereas possible for a digital image analysis software. This shortage of semi-quantitative scoring may result in statistical bias compared to software-based results.

A major difficulty in biological research is the transformation of qualitative data to quantitative data [21]. Semi-quantitative scoring is a widely used method that is able to solve this problem [13,22]. However, we must be aware of its limitations. Subjectivity is a major issue in the scoring process, which is highly influenced by histological expertise [23,24]. In many fields of translational research, tissue scoring is delegated to biomedical personnel (including senior researchers, post-docs and even students) who do not have the same amount of experience as board-certified pathologists, who receive many years of tissue interpretation training. Studies following a ‘do-it-yourself’ pathology approach may suffer from Type I (false positivity) and Type II (false negativity) errors [21]. Although, board-certified pathologists are highly skilled in recognizing patterns in morphological changes, the human visual system has a limited ability to detect subtle changes in tissues, especially with respect to spatial and intensity assessments [22]. A major shortage of conventional scoring and the necessity of a better, higher resolution method is exemplified in Figure 4. Both images were rated with score 2 by all observers, but digital image analysis by Pathronus revealed that the inverse grayscale (0–255 = light to dark) value of first image is 110.02 (Panel A), while of the second is 123.03 (Panel B). This discrepancy arose from the significantly smaller evaluation range (7 vs. 256), resulting in difficulties detecting subtle differences in the labelling intensities with the naked eye and from various human physiological factors such as fatigue and eyestrain, which may occur during the monotonous process of assessing a large number of images [21,25,26]. Although the overall inter-rater agreement was poor, the ranking of mean intensities given to each of the experimental groups was the same for all but one of the observers (Table 3). Better inter-rater agreement might have been reached with a longer pre-training period that was restricted to the evaluation of neuronal cells using this scoring system or by applying cut-offs (e.g., size of neuron or visibility of nuclei). However, it is known from the literature that while high inter-observer agreement is achievable in qualitative scoring (e.g., existence of IHC labelled structures or percentage of positive cells) [27,28,29,30], often fair or poor overall agreement is achieved in the semi-quantitative scoring of staining intensity, even among experts with decades of practice [31]. Generally, results are influenced by study design, the type of tissue being investigated, and how the observers use a specific scoring system [21,22]. Consequently, tissue-specific training with an established scoring system probably improves inter-observer agreement, but it still cannot eliminate the problem of intra-observer variability, such as that observed in the above-detailed physiological factors [22].

Besides inter- and intra-observer reproducibility, it is also essential that the results can be validated. However, as semi-quantitative scoring is the gold standard, digital image analysis of datasets is rarely performed. Studies are not consistent regarding the practicality of the methods that are used. Some authors have reported unequivocal advantages of digital analysis [32], while others did not find any analytical benefits other than time-efficiency [33]. Favorably, a previous quantitative immunofluorescent IHC analysis was carried out on the same cases that were used in the present study, allowing us to make a trustworthy comparison with our current results. Reasonably good agreement was determined in the groups in the order of (CNT > DLB > AD), except for in the case of one investigator. However, the statistically significant differences that were observed among the groups were highly variable, with only the original Pathronus analysis and, perhaps more surprisingly, the converted analysis was able to reproduce the reference data (Table 3). Questions may arise as to why we do not interpret the original Pathronus evaluation as a quantitative technique. CHR-IHC quantification is very difficult due to the numerous variables that are involved from the pre-analytical phase to the post-processing steps, resulting in doubts and inconsistencies in the literature [34,35,36]. Moreover, DAB chromogen does not follow the Beer–Lambert law; the reaction is not stochiometric, and consequently, the staining intensity is not related to the number of antigens [37]. Nonetheless, DAB-based CHR-IHC is the primary choice in diagnostic pathology because it is an easily accessible, relatively cheap, and fast technique compared to quantitative molecular biological methods (i.e., Western blot, qPCR), which may not be feasible in the first-line pathological workup. Furthermore, CHR-IHC intensity-based semiquantitative evaluation is an organic part of several widely used scoring systems with therapeutic relevance (e.g., breast cancer) [8,38]. Originally, the Pathronus platform was developed to support pathologists in routine diagnostic procedures that predominantly required the assessment of CHR-IHC slides. It is an online forum that can be used by pathologists and histologists where they can upload images of difficult or interesting cases and can share and discuss them with other experts from all over the world. In addition, they can teach and train the platform by annotating disease-specific or diagnostically relevant structures on the images. Pathronus may synthesize these data, and the next time somebody uploads a similar image in association with the same disease, the platform may be able to pre-analyze it and label the previously learnt pathologically important ROIs. The software may eliminate inter- and intra-observer bias in the future because the number of investigated neurons is practically unlimited, as the method is able to analyse thousands of cells and can cover whole slides very quickly. However, DAB labelling is still not a quantifiable technique even though results are comparable and can provide a better estimation on protein expression (in accordance with reference datasets) in a standardized experiment (reagents, incubation times, etc.) compared to when the commonly used eyeballing semi-quantitative methods are implemented (Table 3) [16]. Based on the findings outlined here, digital image analysis is undoubtedly the future of histology. Although a comprehensive investigation into the differences between semi-quantitative scoring and digital image analysis was beyond the scope of our current work, an emerging number of publications within this field shed light on a considerable number of features of the two methods. Despite its numerous advantages such as speed, objectivity with good predictive value, its ability to handle large datasets, etc., it also has several disadvantages, namely the cost, equipment requirements, or level of acceptance by some scientific communities and regulators (Table 4) [3,21,22,25,26,33,34,37,39,40,41,42,43,44].

## 5. Conclusions

Semi-quantitative scoring is still widely used as the gold-standard for evaluating CHR-IHC samples. However, it has obvious limitations that need to be addressed. AI-aided software (i.e., Pathronus) might identify cells of interest, differentiate among organelles, protein specific chromogenic labelling, and nuclear counterstaining after an initial training period. This provides a real alternative to semi-quantitative scoring, allowing robust and fast data processing with better predictive value. 

## Figures and Tables

**Table 1:** Confusion matrix on test dataset of the convolutional neural network model trained for differentiation between Class 0 and Class 1 type objects (See Figure 2). n = 907; FN = False Negative; TP = True Positive; TN = True Negative; FP = False Positive.

**Actual Class 1**	21 (FN)	441 (TP)
**Actual Class 0**	388 (TN)	57 (FP)
	**Predicted Class 0**	**Predicted Class 1**

**Table:** 

(**A**)							
CNT		Cohen’s kappa values					
Strength of agreement	Observers	#1	#2	#3	#4	#5	Pathronus
	#1		0.091	0.103	0.6	0.048	−0.01
	#2	poor		0.301	0.195	0.008	−0.012
	#3	poor	fair		0.169	−0.023	−0.009
	#4	moderate	poor	poor		−0.004	−0.034
	#5	poor	poor	poor	poor		0.262
	Pathronus	poor	poor	poor	poor	fair	

**Table:** 

(**B**)							
DLB		Cohen’s kappa values					
Strength of agreement	Observers	#1	#2	#3	#4	#5	Pathronus
	#1		0.063	0.138	0.516	0.226	0.177
	#2	poor		0.316	0.141	0.087	0.048
	#3	poor	fair		0.114	0.143	−0.022
	#4	moderate	poor	poor		0.286	0.196
	#5	fair	poor	poor	fair		0.270
	Pathronus	poor	poor	poor	poor	fair	

**Table:** 

(**C**)							
AD		Cohen’s kappa values					
Strength of agreement	Observers	#1	#2	#3	#4	#5	Pathronus
	#1		0.204	0.179	0.457	0.034	0.195
	#2	poor		0.297	0.285	0.118	0.232
	#3	poor	fair		0.178	0.180	0.062
	#4	moderate	fair	poor		0.260	0.214
	#5	poor	poor	poor	fair		0.116
	Pathronus	poor	fair	poor	fair	poor	

**Table:** 

(**D**)			
	CNT	DLB	AD
Fleiss’ kappa	0.091	0.176	0.183
*p*-value	<0.005	<0.005	<0.005
Agreement	poor	poor	poor

Pairwise inter-rater reliability of semi-quantitative scoring by five observers and converted Pathronus data in CNT (A), DLB (B), and AD (C) groups. Crosstabs contain the Cohen’s kappa values (yellow background) and the strength of agreement (blue background) between two different observers. Sub-table D: Fleiss’kappa values show the overall inter-rater reliability by group and their statistical significance. (CNT = control; DLB = Dementia with Lewy bodies; AD = Alzheimer’s disease).

**Table 3:** Decreasing order of experimental groups based on the mean intensities assessed by semi-quantitative scoring, Pathronus original analysis, and reference data.

Observers	#1	#2	#3	#4	#5	Pathronus Converted	Pathronus Original	Reference Data
Strength of immunopositivity among groups	CNT > DLB > AD	CNT > DLB > AD	CNT > DLB > AD	CNT > DLB > AD	DLB > CNT > AD	CNT > DLB > AD	CNT > DLB > AD	CNT > DLB > AD
Statistical significance (*p* < 0.05)	CNT vs. DLB CNT vs. AD DLB vs. AD	CNT vs. AD	CNT vs. AD	CNT vs. DLB CNT vs. AD DLB vs. AD	-	CNT vs. AD DLB vs. AD	CNT vs. AD DLB vs. AD	CNT vs. AD DLB vs. AD

Strength of immunopositivity is introduced in decreasing order based on mean immunohistochemical (IHC) intensities of the experimental groups, which were determined by the semi-quantitative scoring of five observers, Pathronus original and converted values as well as the immunofluorescent IHC reference method [16]. Statistical significance among groups by analysis of variance (ANOVA) is also presented for every observer and method. (CNT = control; DLB = dementia with Lewy bodies; AD = Alzheimer’s disease; #1–5 = observers).

**Table 4:** Comparison of digital image analysis and semi-quantitative scoring based on relevant factors according to the literature [3,21,22,25,26,33,34,37,39,40,41,42,43,44]. (DAB = 3,3′-Diaminobenzidine).

Digital Image Analysis		Semi-Quantitative Scoring
Expensive	Cost	Cheap
Fast	Speed	Slow
Not required (except training period)	Histological experiment	Required
Objective (with standard settings)	Objectivity	Subjective
Based on software and settings	Inter-rater variability	Considerable
Not applicable	Intra-rater variability	Notable
Yes (except DAB labelling)	Quantification	Not applicable
Automatic (after training period)	Operation	Manual
Large	Data volume	Limited
IT background, slide scanner	Equipment	Light microscope
New era	Research purposes	Gold standard

## Data Availability

The datasets used and/or analysed during the current study are available from the corresponding author upon reasonable request.

## Supplementary Materials

The following supporting information can be downloaded at: https://www.mdpi.com/article/10.3390/biom12010019/s1, Supplementary Table S1. Table shows the semi-quantitative immunohistochemical intensity scores given by five observers as well as the original and converted Pathronus data for each image categorized by cases and groups. Mean intensities/cases are also included. (CNT = control; DLB = dementia with Lewy bodies; AD = Alzheimer’s disease; #1–5 = observers]

## References

[1] Capobianco E., Iacoviello L., de Gaetano G., Donati M.B. (2020). Editorial: Trends in Digital Medicine. Front. Med..

[2] Van Timmeren J.E., Cester D., Tanadini-Lang S., Alkadhi H., Baessler B. (2020). Radiomics in Medical Imaging—“How-to” Guide and Critical Reflection. Insights Imaging.

[3] Kayser K., GĂśrtler J., Bogovac M., Bogovac A., Goldmann T., Vollmer E., Kayser G. (2010). AI (Artificial Intelligence) in Histopathology--from Image Analysis to Automated Diagnosis. Folia Histochem. Cytobiol..

[4] Csonka T., Murnyák B., Szepesi R., Bencze J., Bognár L., Klekner Á., Hortobágyi T. (2016). Assessment of Candidate Immunohistochemical Prognostic Markers of Meningioma Recurrence. Folia Neuropathol..

[5] Hortobágyi T., Bencze J., Varkoly G., Kouhsari M.C., Klekner Á. (2016). Meningioma Recurrence. Open Med..

[6] Bencze J., Szarka M., Bencs V., Szabó R.N., Módis L.V., Aarsland D., Hortobágyi T. (2020). Hortobágyi Lemur Tyrosine Kinase 2 (LMTK2) Level Inversely Correlates with Phospho-Tau in Neuropathological Stages of Alzheimer’s Disease. Brain Sci..

[7] Crowe A., Yue W. (2019). Semi-Quantitative Determination of Protein Expression Using Immunohistochemistry Staining and Analysis: An Integrated Protocol. Bio-Protocol.

[8] Hanna W., O’Malley F.P., Barnes P., Berendt R., Gaboury L., Magliocco A., Pettigrew N., Robertson S., Sengupta S., Têtu B. (2007). Updated Recommendations from the Canadian National Consensus Meeting on HER2/Neu Testing in Breast Cancer. Curr. Oncol..

[9] Attems J., Toledo J.B., Walker L., Gelpi E., Gentleman S., Halliday G., Hortobagyi T., Jellinger K., Kovacs G.G., Lee E.B. (2021). Neuropathological Consensus Criteria for the Evaluation of Lewy Pathology in Post-Mortem Brains: A Multi-Centre Study. Acta Neuropathol..

[10] Kovacs G.G., Xie S.X., Lee E.B., Robinson J.L., Caswell C., Irwin D.J., Toledo J.B., Johnson V.E., Smith D.H., Alafuzoff I. (2017). Multisite Assessment of Aging-Related Tau Astrogliopathy (ARTAG). J. Neuropathol. Exp. Neurol..

[11] Alafuzoff I., Thal D.R., Arzberger T., Bogdanovic N., Al-Sarraj S., Bodi I., Boluda S., Bugiani O., Duyckaerts C., Gelpi E. (2009). Assessment of β-Amyloid Deposits in Human Brain: A Study of the BrainNet Europe Consortium. Acta Neuropathol..

[12] Módis L.V., Varkoly G., Bencze J., Hortobágyi T.G., Módis L., Hortobágyi T. (2021). Extracellular Matrix Changes in Corneal Opacification Vary Depending on Etiology. Mol. Vis..

[13] Walker R.A. (2006). Quantification of Immunohistochemistry—Issues Concerning Methods, Utility and Semiquantitative Assessment I. Histopathology.

[14] Deo R.C. (2015). Machine Learning in Medicine. Circulation.

[15] Komura D., Ishikawa S. (2018). Machine Learning Methods for Histopathological Image Analysis. Comput. Struct. Biotechnol. J..

[16] Bencze J., Szarka M., Bencs V., Szabó R.N., Smajda M., Aarsland D., Hortobágyi T. (2019). Neuropathological Characterization of Lemur Tyrosine Kinase 2 (LMTK2) in Alzheimer’s Disease and Neocortical Lewy Body Disease. Sci. Rep..

[17] Skogseth R., Hortobágyi T., Soennesyn H., Chwiszczuk L., Ffytche D., Rongve A., Ballard C., Aarsland D. (2017). Accuracy of Clinical Diagnosis of Dementia with Lewy Bodies versus Neuropathology. J. Alzheimer’s Dis..

[18] Vitrolink An Online Digital Image Analysis Platform. https://vitrolink.com/#/products.

[19] Lin T.-Y., Goyal P., Girshick R., He K., Dollar P. (2020). Focal Loss for Dense Object Detection. IEEE Trans. Pattern Anal. Mach. Intell..

[20] Landis J.R., Koch G.G. (1977). The Measurement of Observer Agreement for Categorical Data. Biometrics.

[21] Meyerholz D.K., Beck A.P. (2018). Fundamental Concepts for Semiquantitative Tissue Scoring in Translational Research. ILAR J..

[22] Meyerholz D.K., Beck A.P. (2018). Principles and Approaches for Reproducible Scoring of Tissue Stains in Research. Lab. Investig..

[23] Gavrielides M.A., Gallas B.D., Lenz P., Badano A., Hewitt S.M. (2011). Observer Variability in the Interpretation of HER2/*Neu* Immunohistochemical Expression With Unaided and Computer-Aided Digital Microscopy. Arch. Pathol. Lab. Med..

[24] Cross S.S., Betmouni S., Burton J.L., Dubé A.K., Feeley K.M., Holbrook M.R., Landers R.J., Lumb P.B., Stephenson T.J. (2000). What Levels of Agreement Can Be Expected Between Histopathologists Assigning Cases to Discrete Nominal Categories? A Study of the Diagnosis of Hyperplastic and Adenomatous Colorectal Polyps. Mod. Pathol..

[25] Aeffner F., Wilson K., Martin N.T., Black J.C., Hendriks C.L.L., Bolon B., Rudmann D.G., Gianani R., Koegler S.R., Krueger J. (2017). The Gold Standard Paradox in Digital Image Analysis: Manual Versus Automated Scoring as Ground Truth. Arch. Pathol. Lab. Med..

[26] Rizzardi A.E., Johnson A.T., Vogel R.I., Pambuccian S.E., Henriksen J., Skubitz A.P., Metzger G.J., Schmechel S.C. (2012). Quantitative Comparison of Immunohistochemical Staining Measured by Digital Image Analysis versus Pathologist Visual Scoring. Diagn. Pathol..

[27] Jasani B., Bänfer G., Fish R., Waelput W., Sucaet Y., Barker C., Whiteley J.L., Walker J., Hovelinck R., Diezko R. (2020). Evaluation of an Online Training Tool for Scoring Programmed Cell Death Ligand-1 (PD-L1) Diagnostic Tests for Lung Cancer. Diagn. Pathol..

[28] Pang J.M.B., Castles B., Byrne D.J., Button P., Hendry S., Lakhani S.R., Sivasubramaniam V., Cooper W.A., Armes J., Millar E.K.A. (2021). SP142 PD-L1 Scoring Shows High Interobserver and Intraobserver Agreement in Triple-Negative Breast Carcinoma but Overall Low Percentage Agreement with Other PD-L1 Clones SP263 and 22C3. Am. J. Surg. Pathol..

[29] Chang S., Park H.K., Choi Y.L., Jang S.J. (2019). Interobserver Reproducibility of PD-L1 Biomarker in Non-Small Cell Lung Cancer: A Multi-Institutional Study by 27 Pathologists. J. Pathol. Transl. Med..

[30] Cooper W.A., Russell P.A., Cherian M., Duhig E.E., Godbolt D., Jessup P.J., Khoo C., Leslie C., Mahar A., Moffat D.F. (2017). Intra- and Interobserver Reproducibility Assessment of PD-L1 Biomarker in Non–Small Cell Lung Cancer. Clin. Cancer Res..

[31] Lidbury J.A., Rodrigues Hoffmann A., Ivanek R., Cullen J.M., Porter B.F., Oliveira F., van Winkle T.J., Grinwis G.C., Sucholdolski J.S., Steiner J.M. (2017). Interobserver Agreement Using Histological Scoring of the Canine Liver. J. Vet. Intern. Med..

[32] Rizzardi A.E., Zhang X., Vogel R.I., Kolb S., Geybels M.S., Leung Y.-K., Henriksen J.C., Ho S.-M., Kwak J., Stanford J.L. (2016). Quantitative Comparison and Reproducibility of Pathologist Scoring and Digital Image Analysis of Estrogen Receptor Β2 Immunohistochemistry in Prostate Cancer. Diagn. Pathol..

[33] Ong C.W., Kim L.G., Kong H.H., Low L.Y., Wang T.T., Supriya S., Kathiresan M., Soong R., Salto-Tellez M. (2010). Computer-Assisted Pathological Immunohistochemistry Scoring Is More Time-Effective than Conventional Scoring, but Provides No Analytical Advantage. Histopathology.

[34] Taylor C.R., Levenson R.M. (2006). Quantification of Immunohistochemistry?Issues Concerning Methods, Utility and Semiquantitative Assessment II. Histopathology.

[35] Cregger M., Berger A.J., Rimm D.L. (2006). Immunohistochemistry and Quantitative Analysis of Protein Expression. Arch. Pathol. Lab. Med..

[36] di Cataldo S., Ficarra E., Macii E. (2012). Computer-Aided Techniques for Chromogenic Immunohistochemistry: Status and Directions. Comput. Biol. Med..

[37] van der Loos C.M. (2008). Multiple Immunoenzyme Staining: Methods and Visualizations for the Observation with Spectral Imaging. J. Histochem. Cytochem..

[38] Wolff A.C., Hammond M.E.H., Schwartz J.N., Hagerty K.L., Allred D.C., Cote R.J., Dowsett M., Fitzgibbons P.L., Hanna W.M., Langer A. (2007). American Society of Clinical Oncology/College of American Pathologists Guideline Recommendations for Human Epidermal Growth Factor Receptor 2 Testing in Breast Cancer. Arch. Pathol. Lab. Med..

[39] Pell R., Oien K., Robinson M., Pitman H., Rajpoot N., Rittscher J., Snead D., Verrill C. (2019). The Use of Digital Pathology and Image Analysis in Clinical Trials. J. Pathol. Clin. Res..

[40] Aeffner F., Zarella M.D., Buchbinder N., Bui M.M., Goodman M.R., Hartman D.J., Lujan G.M., Molani M.A., Parwani A.V., Lillard K. (2019). Introduction to Digital Image Analysis in Whole-Slide Imaging: A White Paper from the Digital Pathology Association. J. Pathol. Inform..

[41] Farahani N., Parwani A.V., Pantanowitz L. (2015). Whole Slide Imaging in Pathology: Advantages, Limitations, and Emerging Perspectives. Pathol. Lab. Med. Int..

[42] Shrestha P., Kneepkens R., van Elswijk G., Vrijnsen J., Ion R., Verhagen D., Abels E., Vossen D., Hulsken B., Shrestha P. (2015). Objective and Subjective Assessment of Digital Pathology Image Quality. AIMS Med. Sci..

[43] Tadrous P.J. (2010). On the Concept of Objectivity in Digital Image Analysis in Pathology. Pathology.

[44] Eggerschwiler B., Canepa D.D., Pape H.C., Casanova E.A., Cinelli P. (2019). Automated Digital Image Quantification of Histological Staining for the Analysis of the Trilineage Differentiation Potential of Mesenchymal Stem Cells. Stem Cell Res. Ther..

